# DDQR (dynamic DNA QR coding): An efficient algorithm to represent DNA barcode sequences

**DOI:** 10.1371/journal.pone.0279994

**Published:** 2023-01-17

**Authors:** Yujun Wang, Xinjing Yao, Rui Liu, Chang Liu

**Affiliations:** 1 School of Information Management, Central China Normal University, Wuhan, Hubei, P.R. China; 2 Institute of Medicinal Plant Development, Chinese Academy of Medical Science, Beijing, P.R. China; Tierarztliche Hochschule Hannover, GERMANY

## Abstract

A DNA barcode is a short piece of standard DNA sequence used for species determination and discrimination. Representation of DNA barcodes is essential for DNA barcodes’ applications in the transportation and recognition of biological materials. Previously, we have compared different strategies for representing the DNA barcodes. In the present study, we have developed a compression algorithm based on binary coding or Huffman coding scheme, followed by converting the binary digits into Base64 digits. The combination of this compression algorithm and the QR representation leads to the dynamic DNA QR coding algorithm (DDQR). We tested the DDQR algorithm on simulated data and real DNA barcode sequences from the commonly used plant and animal DNA barcode markers: rbcL, matK, trnH-psbA, ITS2, and COI. We compared the compression efficiency of DDQR and another state-of-the-art DNA compression algorithm GeCo3 for sequences with various base compositions and lengths. We found that DDQR had a higher compression rate than GeCo3 for DNA sequences shorter than 800 bp, which is the typical size range for DNA barcodes. We also upgraded a web server (http://www.1kmpg.cn/ddqr) that provides three functions: retrieval of DNA barcode sequences, encoding DNA barcode sequences to DDQR codes, and decoding DDQR codes to DNA barcode sequences. The DDQR algorithm and the webserver will be invaluable to applying DNA barcode technology in the food and traditional medicine industries.

## 1. Introduction

Traditional medicines have been and still are widely used in different countries. For example, in China alone, the estimated annual product sales of goods related to traditional medicines approach 100 billion $US. One of the main problems facing the traditional medicine industry is the difficulty in product quality control. In particular, mislabeling the biological compositions posed a significant threat to the product’s efficacy and safety.

DNA barcode technology has been developed for identifying biological materials for the past several years. For example, ITS2 and *psbA-trnH* have been developed to determine the identity of the plant composition for those plant species listed in the Chinese pharmacopeia [1]. To check if the samples’ identity is consistent with those on the products’ labels, one approach is associating the DNA barcodes with the labels for the physical products. Samples of the physical products can be taken at any point of the production and supply chain for a biological assay. Its identity can be determined using DNA barcoding technology and then compared with those on the labels.

Our previous study showed that QR code performed better than other barcode schemes to encode DNA barcode sequences [2]. However, the DNA barcode sequences are too long, resulting in a QR code too big for practical use. In the present study, we develop an algorithm that will compress the DNA barcode sequences for biological materials to generate a QR code of reasonable size.

### 1.1 DNA barcoding technology

A DNA barcode is a DNA sequence that originated from the genome of a species, and it has been developed as a marker to distinguish a focal species from other species [3]. Several DNA barcode markers have been developed and widely used. For animals, the Mitochondrial cytochrome C oxidase subunit 1 (COI) gene is the most commonly used DNA barcode sequence [4, 5]. For plants, it was found earlier that a single DNA locus has limited resolving power for closely related species after comparing several coding and non-coding sequences [6, 7]. Later, the Consortium for Barcode of Life (CBOL) plant working group proposed to use *matK* and *rbcL* as plant barcode markers. This recommendation was made after comparing several candidate markers based on the following three criteria. Firstly, the marker should be amplified easily with a single primer pair. Secondly, the maker should be amenable to bidirectional sequencing with little manual editing. And lastly, the marker sequence should have high resolving power in species discrimination [8].

In addition to *matK* and *rbcL*, the intergenic transcribed spacer (ITS) and its subsequence (ITS2) were proposed as additional core barcodes [2], and the nuclear ribosomal internal transcribed spacer (ITS) region was chosen as a universal DNA barcode marker for Fungi [9]. Furthermore, *psbA-trnH* remained a supplementary DNA barcode and a promising candidate for efficient identification [2]. As a result, COI for animals and *rbcL*, *matK*, ITS2, and *psbA-trnH* for plants are the best markers to determine the identity of materials used in traditional medicines.

Previously, we compared the representation of DNA barcode sequences using various coding schemes [2]. The results showed that the QR code has the most efficient representation. However, due to the size of the DNA barcode, the QR code can be too big to be used in a real-world application. As a result, an additional compression algorithm is in urgent need.

### 1.2 Different methods for DNA sequence compression

Previous research work has developed several methods for compressing DNA sequences. For instance, GReEn is an efficient tool to compress genome resequencing data [10]. It used a probabilistic copy model for compression. The probabilities were estimated for every base of the focal sequence and fed to an arithmetic coder. The are two control parameters for GReEn. The first one is the size of the k-mer used for searching copies. And the second one is the number of prediction failures tolerated by the copy model. In contrast, GeCo3 used a neural network to compress a genomic sequence, gaining extra compression over other state-of-the-art compressors. GeCo3 took advantage of a portable mixing method. It only required the model probabilities as inputs. As a result, it could adapt to other data compression tools [11]. It improved compression significantly over the previous version (GeCo2). The method has been tested on reference-based and reference-free compression applications and demonstrated significant improvement over the earlier methods. The highest compression rate was obtained while a reference was used.

DNA barcode sequences were designed for species determination and discrimination across a wide range of the taxonomic group. However, the DNA barcode sequences are not suitable for reference-based methods. The sequences derived from taxonomic groups would dramatically vary in sequence length and composition. And the selection of a reference sequence would be impractical. As a result, a reference-free compression algorithm is needed to compress the DNA barcode.

We developed a Dynamic DNA QR coding algorithm (DDQR) to solve this problem in the present study. The algorithm was superior to the GeCo3 algorithm while processing shorter DNA sequences using simulated and real data at various compositions and lengths. Lastly, a web server was developed, allowing the easy use of the algorithm. DDQR code has a wide range of applications. In combination with technologies such as PCR and Sanger sequencing, DDQR can be used to determine the authenticity of biological materials. On the other hand, the entire production chain can be traced in combination with the time and location information collected whenever the DDQR code is scanned. Details on the applications were described in the discussion sections.

## 2. Materials and methods

### 2.1 Algorithm

This DDQR algorithm increases the capacity of the QR code to store DNA sequences by dynamically selecting the coding schemes. The following is a detailed description of this dynamic coding method.

Given a DNA sequence composed of four characters [’A,’ ’G,’ ’C,’ ’T’], two steps were taken to compress the original sequences. Firstly, the sequences were converted to a string of binary digits. Secondly, the binary string was converted to a Base64 digit string. In the first step, two different coding schemes were compared. We calculated the frequencies of the four nucleotides (nt), assuming the frequencies of the four nucleotides follow the order nt1 ≥ nt2 ≥ nt3 ≥ nt4. The first scheme was a simple binary coding. For example, we used the following coding table: 00->nt1, 01->nt2, 10->nt3, 11->nt4. The coding method can be seen in Table 1. The second scheme was the Huffman coding [12]. We then used the following coding table, ’1’->nt1, ’01’->nt2, ’001’->nt3 and ’000’->nt4. The coding method can be seen in Table 1.

**Table 1:** Coding scheme for DDQR code.

Two bit coding	Huffman coding
nt1: 00	nt1: 1
nt2: 01	nt2: 01
nt3: 10	nt3: 000
nt4: 11	nt4: 001

Here nt: nucleotide, can be “A”, “G”, “C” and “T”.

In particular, the length of the binary digits coded with the binary table was two times the original length. In contrast, the length of the binary digits coded with the Huffman table were 1*freq(nt1)+2*freq(nt1)+3*(freq(nt3)+freq(nt4)) times the original length. Here freq(nt) represents the frequency of the corresponding nucleotide.

Then, the length of the resulting string of binary coding and the Huffman coding scheme were compared. The one with a smaller size was chosen to be converted to a string of Base64 digits. Base64 is the most popular binary-to-text algorithm to convert binary data into plain text to prevent data corruption during transmission. Before the conversion, we added tags’ 000’ or ’111’ to the beginning of the binary string to represent Huffman coding and binary coding, respectively. The bit string obtained was converted into a Base64 digit every six bits. If the number of the last group of digits was less than six, we used ’0’ to make it up to six digits.

### 2.2 Preparation of simulated data

Simulated sequences of 1000 bp long were generated using a custom Python script. We assumed that the base ’A’ had the most significant proportion, and its proportions increased from 25% to 95% by an increment of 1% each time. The proportions of the remaining bases’ T’, ’C,’ and ’G’ were generated randomly.

### 2.3 Preparation of real data

As described earlier, four plant DNA barcodes (ITS2, *rbcL*, *matK* and *psbA*-*trnH*) and one animal DNA barcode (*CO1*) have been well developed. As a result, we tested the sequences of these five markers with our algorithm. We searched GenBank with the keywords corresponding to the DNA barcode markers. Then we downloaded the corresponding GenBank records for the DNA barcode sequences identified. The sequences in the FASTA format for the four plant DNA barcodes were parsed using custom Perl scripts. The *CO1* sequences in the GenBank have a particular “barcode” tag. As a result, the *CO1* sequences were retrieved from GenBank (Version 188) with the keyword "barcode".

There was a more significant number of the *CO1* sequences than other markers. Using all *CO1* sequences in the test became prohibitively expensive. Thus, we selected the *CO1* sequences for Lepidoptera (butterflies and moths) for the test.

According to the preliminary analysis, this dataset’s length distribution and sequence composition were comparable to those of the dataset containing all *CO1* sequences.

After the test sequences were collected, they were subjected to pre-processing, including the following steps. Firstly, we checked the downloaded sequences for their orientations. Secondly, we removed the flanking sequences that were not considered part of the DNA barcode sequences. Thirdly, we removed the sequences that had unusual lengths. We kept those sequences between 150 and 600 bp long for the four plant barcodes. For *CO1* barcodes, we kept the sequences 100 and 700 bp long for the analysis.

### 2.4 Web server construction

The DDQR algorithms were implemented using the JAVA language. The source code for DDQR and GeCo3 compression rate calculation is provided in S6 File. The source code for DDQR compression rate calculation using simulated data is provided in S7 File. The source code for GeCo3 compression rate calculation using the simulated data is provided in S8 File. The source code for DDQR compression rate calculation using the real data is provided in S9 File. The source code for GeCo3 compression rate calculation using the real data is provided in S10 File.

The DDQR web server application was developed using the Perl Catalyst Framework (5.16) on an Apache server (2.2.14) running on a Centos 7.6 Linux operating system. The web server has been tested on the Windows platform using Microsoft Edge (versions 78, 79, and 80), Firefox (versions 36, 37, and 38), and the Mac platform using Safari (versions 5.0, and 5.1).

## 3. Results

### 3.1 Performance of the DDQR using simulated data

#### 3.1.1 Comparison of the compression rates of DDQR and GeCo3 using simulated data

The most important factors affecting the two algorithms’ performances include the DNA sequence’s base composition and length. As a result, we compared the two algorithms under various conditions for these two factors. Firstly, we intended to identify the general trend of the compression rates for the two algorithms. The composition of a particular type of nucleotide was set from 20% to 100% with a step of 1%. The DNA sequence length was set from 1 to 1500 bp. Five hundred sequences were generated at each base composition and each sequence length. And each sequence was subjected to analysis by DDQR and GeCo3, respectively. We calculated the string length after the DDQR processing divided by the length of the input DNA sequence (compression rate). The compression rates had averages of 0.345 (length = 200 bp), 0.34 (length = 300 bp) and 0.3375 (length = 400 bp).

**Fig 1:**
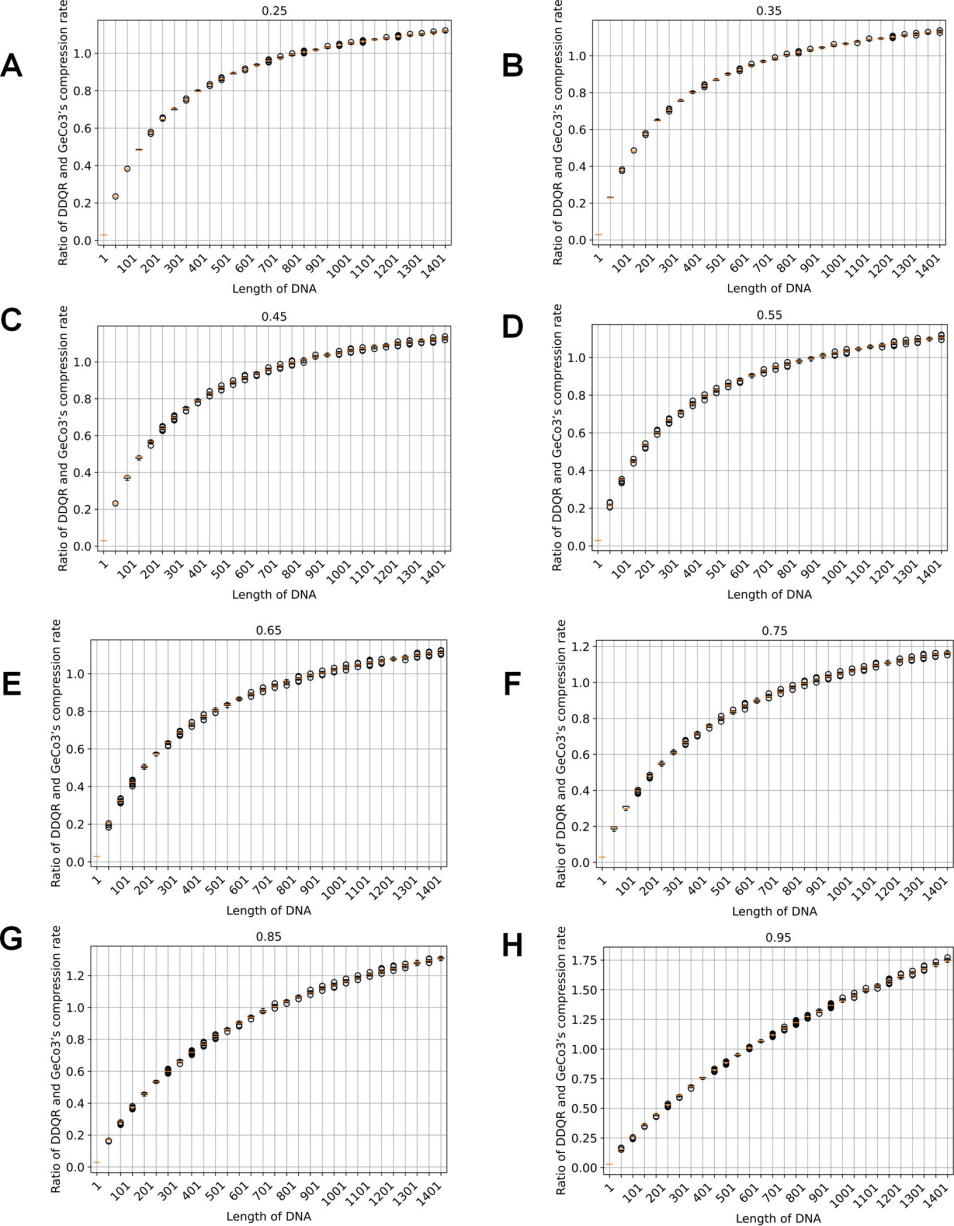
Comparison of compression rates of DDQR and Geco3 using simulated data. The panels represent results obtained for DNA sequences having a particular composition: 25% (A), 35% (B), 45% (C), 55% (D), 65% (E), 75% (F), 85% (G), 95% (H). The x-axis is the DNA sequence length. The y-axis is the ratio of DDQR and GeCo3’s compression rates.

We calculated the ratio of DDQR and GeCo3’s compression rates and plotted the ratios against the corresponding base compositions. The results are shown in Fig 1. The DDQR is more efficient than the GeCo3 algorithm when the sequence size is small. However, when the sequence size increased, the ratio of DDQR and GeCo3’s compression rate increased and eventually exceeded 1.

**Fig 2:**
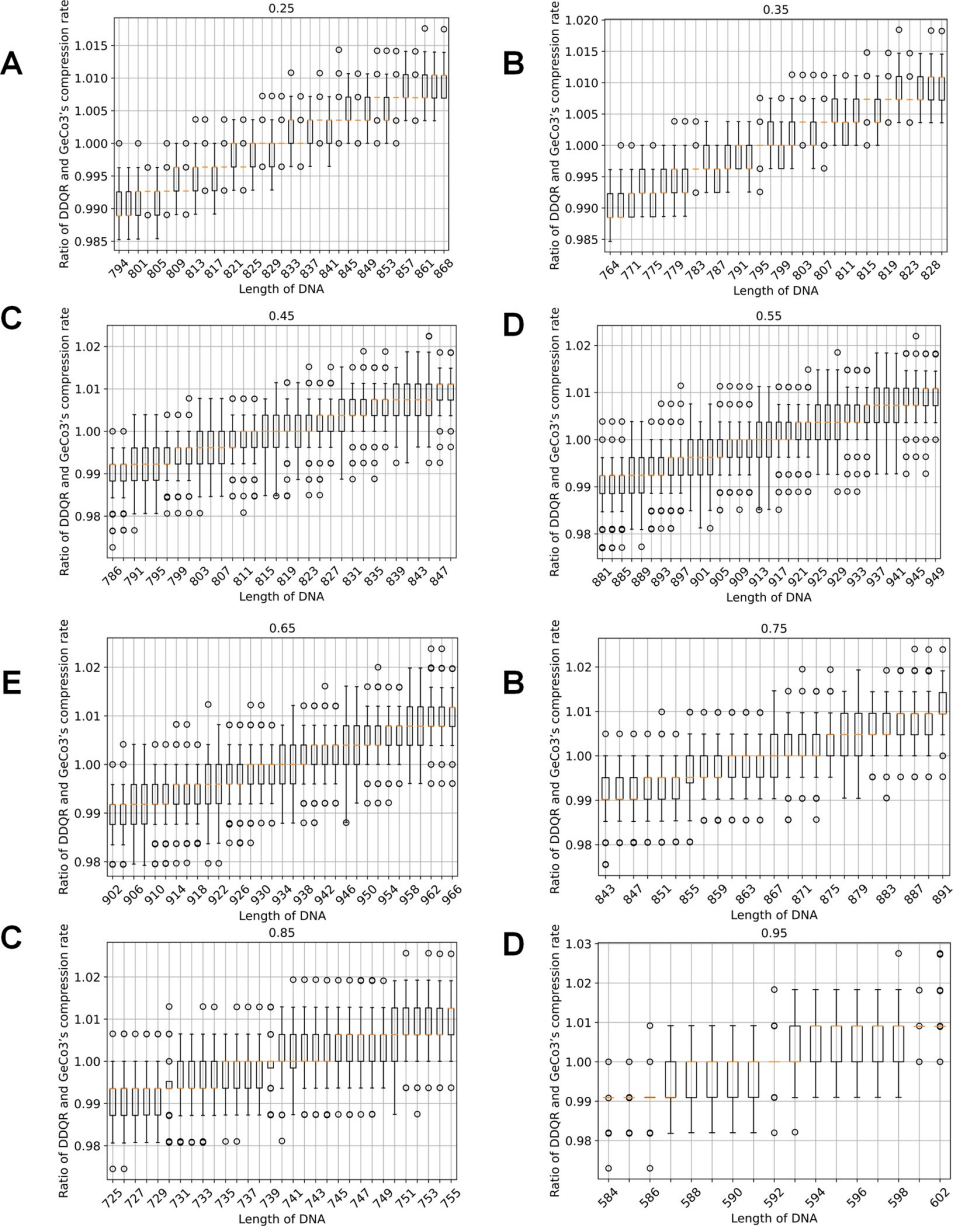
Comparison of compression rates of DDQR and Geco3 using simulated data around the region where the performance of DDQR exceeds that of GeCo3. The panels represent results obtained for DNA sequences having a particular composition: 25% (A), 35% (B), 45% (C), 55% (D), 65% (E), 75% (F), 85% (G), 95% (H). The x-axis is the DNA sequence length. The y-axis is the ratio of DDQR and GeCo3’s compression rates.

We also plotted out the regions around which the ratio of DDQR and GeCo3’s compression rate exceeded 1, indicating that the DDQR’s performance became poorer than GeCo3. As shown in Fig 2, the regions ranging from 821–841 (25%, a particular type of nucleotide composition), 785–811 (35%), 805–832 (45%), 899–929 (55%), 920–948 (65%), 855–879 (75%), 731–750 (85%) and 587–598 (95%). The percentage in the parenthesis represents the fixed composition of one nucleotide.

#### 3.1.2 Identification of the cutoff length for DDQR

**Fig 3:**
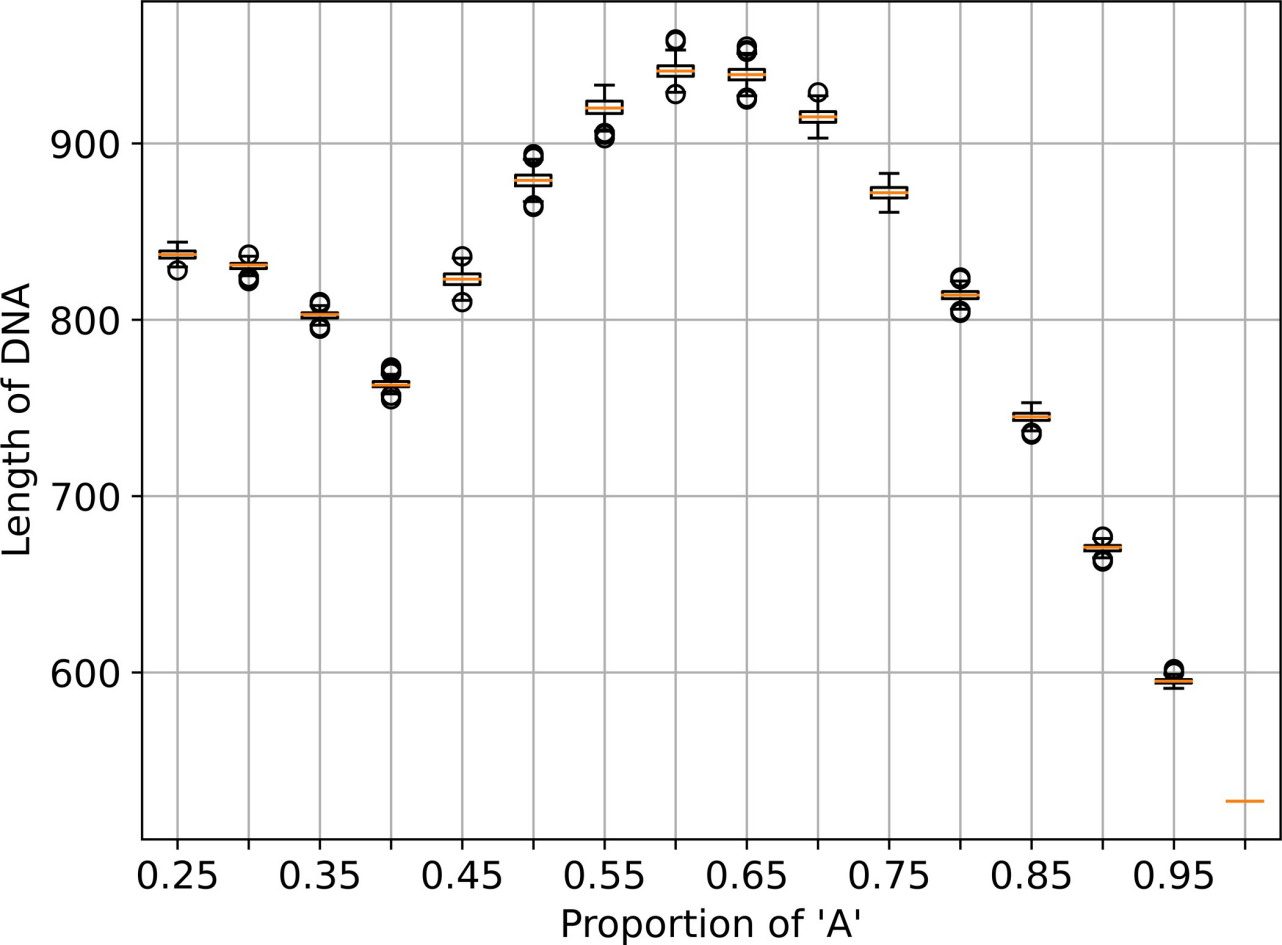
The length threshold of DNA sequence for successful compression using DDQR. The x-axis shows the DNA compositions. The y-axis shows the length of DNA sequences.

After we identified the general trend of the compression rate, we selected the regions where the ratio of the compression rates is close to 1 for detailed characterization. We generated sequences with the composition of a particular type of nucleotide varied from 25%-100%, with a step of 1%. At each composition, we generated sequences having lengths (i) starting from i = 1 to i = 1000. At each length i, we calculated the ratio of DDQR and GeCo3’s compression rate. To determine the exact length at which the ratio of DDQR and GeCo3’s compression rate exceeded 1. We adopted a sliding window approach. The size of the sliding window was set to 10. The sliding window started from a sequence length equivalent to (i-10) and ended with the current sequence length i. The sliding window had 11 ratios corresponding to the length ranging from (i-10) to i. We counted the numbers of these eleven ratios that were greater than 1. Whenever 6 out of the 11 ratios were greater than 1, we determined the (i-5) cutoff length for DDQR to be superior to GeCo3. We found the threshold to be 837.172 (25%), 802.722 (35%), 823.146 (45%), 920.14 (55%), 938.891 (65%), 872.156 (75%), 744.695 (85%), and 595.408 (95%). The results are shown in Fig 3.

### 3.2 Performance of DDQR using real data

#### 3.2.1 Compression rate of DDQR using real data

**Fig 4:**
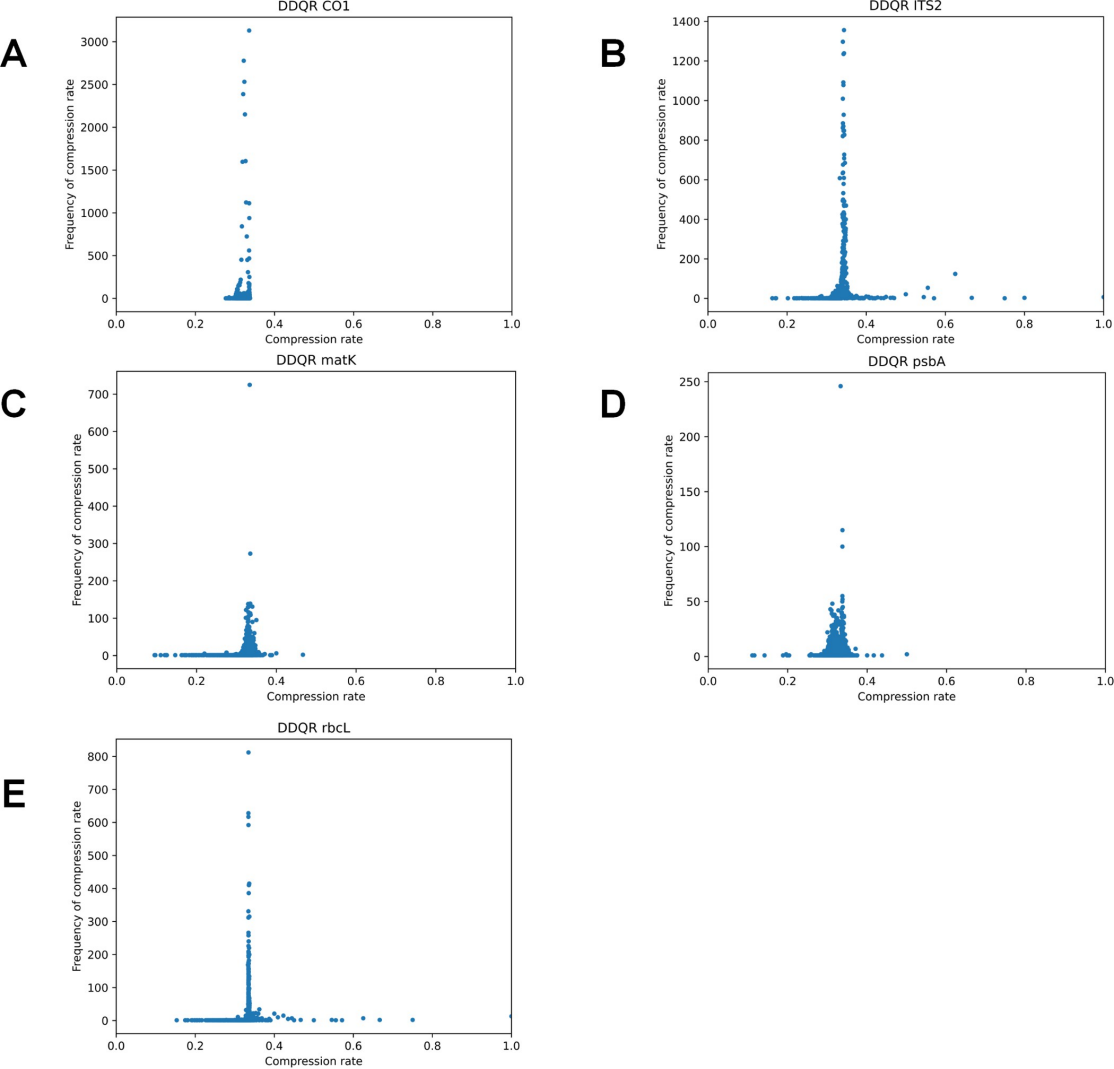
Compression rate of DDQR using real data. Five different DNA barcode marker datasets are used: ITS2 (A), rbcL(B), matK (C), psbA-trnH (D), and CO1(E). The X-axis is the compression rate. The Y-axis is the frequency of the corresponding rate.

Five sets of real data, ITS2, *rbcL*, *matK*, *psbA-trnH*, and *CO1*, were used to test the compression efficiency of the DDQR algorithm using the same method described above. The results are shown in Fig 4. The following five graphs respectively show the compression rate distribution of DNA sequences in each data set. The horizontal coordinates indicate the compression rate, and the vertical coordinates indicate the corresponding frequencies.

The data set for DNA barcode *rbcL* contained 29,752 DNA sequences (S1 File). The DNA sequence contained an average of 1184 bases. The average compression rate for this data set was 33.51% (Fig 4A). The data set for *matK* contained 28,972 DNA sequences (S2 File), and each DNA sequence contained an average of 1200 bases. The average compression rate for this data set was 32.89% (Fig 4B). The data set for *psb*A-*trn*H contained 13,634 DNA sequences (S3 File), and each DNA sequence contained an average of 393 bases. The average compression rate for this data set was 32.50% (Fig 4C). The data set for ITS2 contained 62,303 DNA sequences (S4 File). Each DNA sequence contained an average of 219 bases. The average compression rate for this dataset was 34.26% (Fig 4D). The data set for *CO1* contained 31,783 DNA sequences (S5 File). Each DNA sequence contained an average of 659 bases. The average compression rate for this data set was 32.65% (Fig 4E).

#### 3.2.2 Comparison of the compression rates of DDQR and GeCo3 using real data

**Fig 5:**
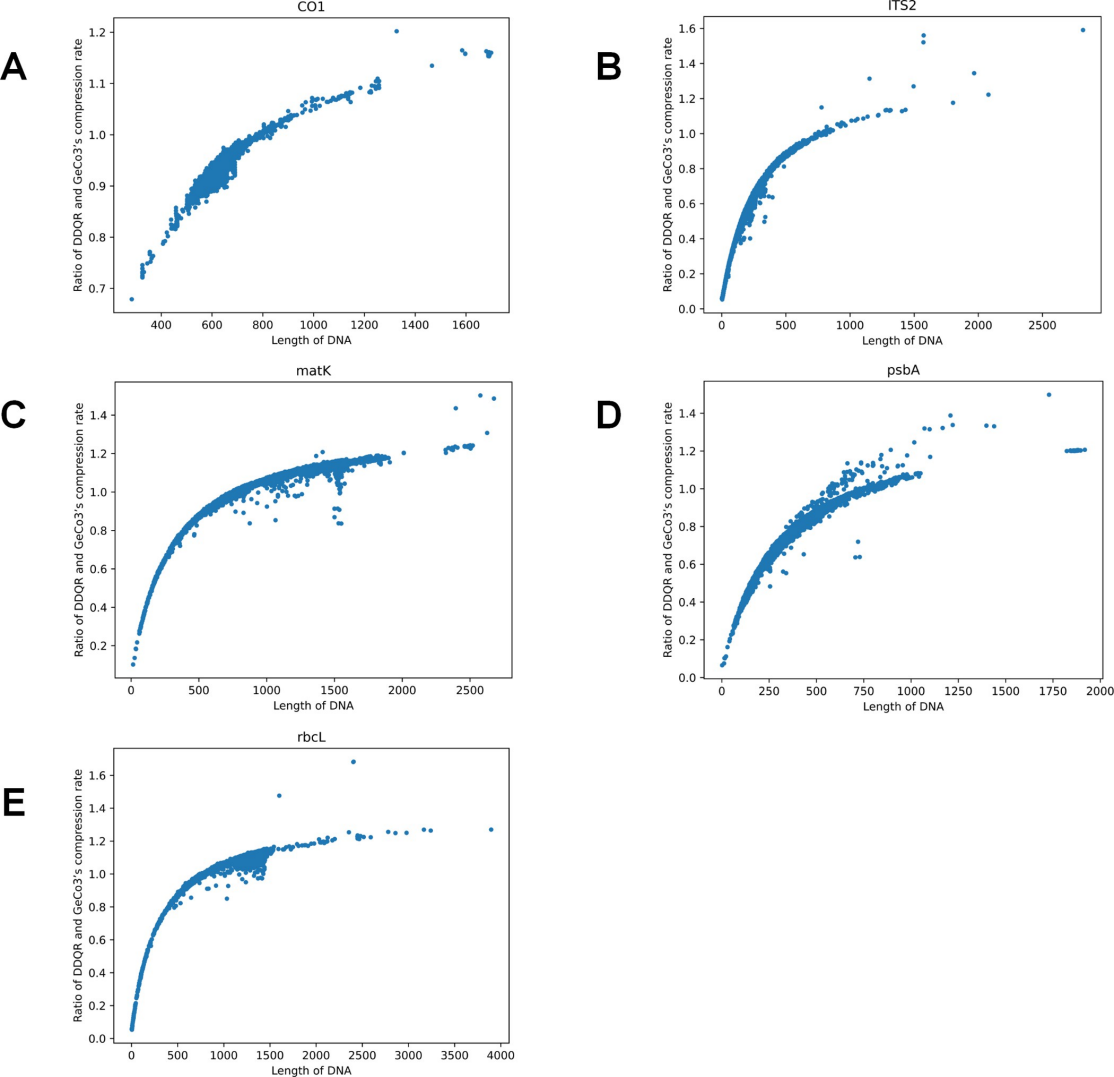
Comparison of compression rates of DDQR and Geco3 using real data. Five different DNA barcode marker datasets are used: ITS2 (A), rbcL(B), matK (C), psbA-trnH (D), and CO1 (E). The X-axis is the compression rate. The Y-axis is the frequency of the corresponding ratio of DDQR and GeCo3’s compression rates.

We analyzed the ratio of the compression rates of DDQR and GeCo3 using the five real data sets for ITS2, *rbcL*, *matK*, *psbA-trnH*, and *CO1* described above. The results are shown in Fig 5. The ratio of the compression rates of two different algorithms showed similar overall patterns. However, some minor differences can be seen. The DDQR algorithm proved to have better performance when processing shorter DNA sequences (sequence length ≤ 800).

## 4. Webserver structure and function

A web server was implemented that allows the users to retrieve DDQR code for the five most popular DNA barcode markers, including ITS2, *rbcL*, *matK*, *psbA-trnH*, and *CO1* (http://www.1kmpg.cn/ddqr). The DDQR web server is an upgrade of our previous published work on the QR server [2]. It contains three modules. Module 1 (Fig 6.1) allows users to retrieve the barcode sequences of ITS2, *rbcL*, *matK*, *psbA-trnH*, and *CO1* for a focal species by Latin name or taxid. Module 2 (Fig 6.2) is the encoder of the DDQR algorithm. It takes a DNA sequence as the input (Fig 6.2A) and returns the encoded DDQR code (Fig 6.2B). Module 3 (Fig 6.3) is the decoder of the DDQR algorithm. It takes a DDQR code as input (Fig 6.3A) and decodes it into the DNA sequence (Fig 6.3B).

**Fig 6:**
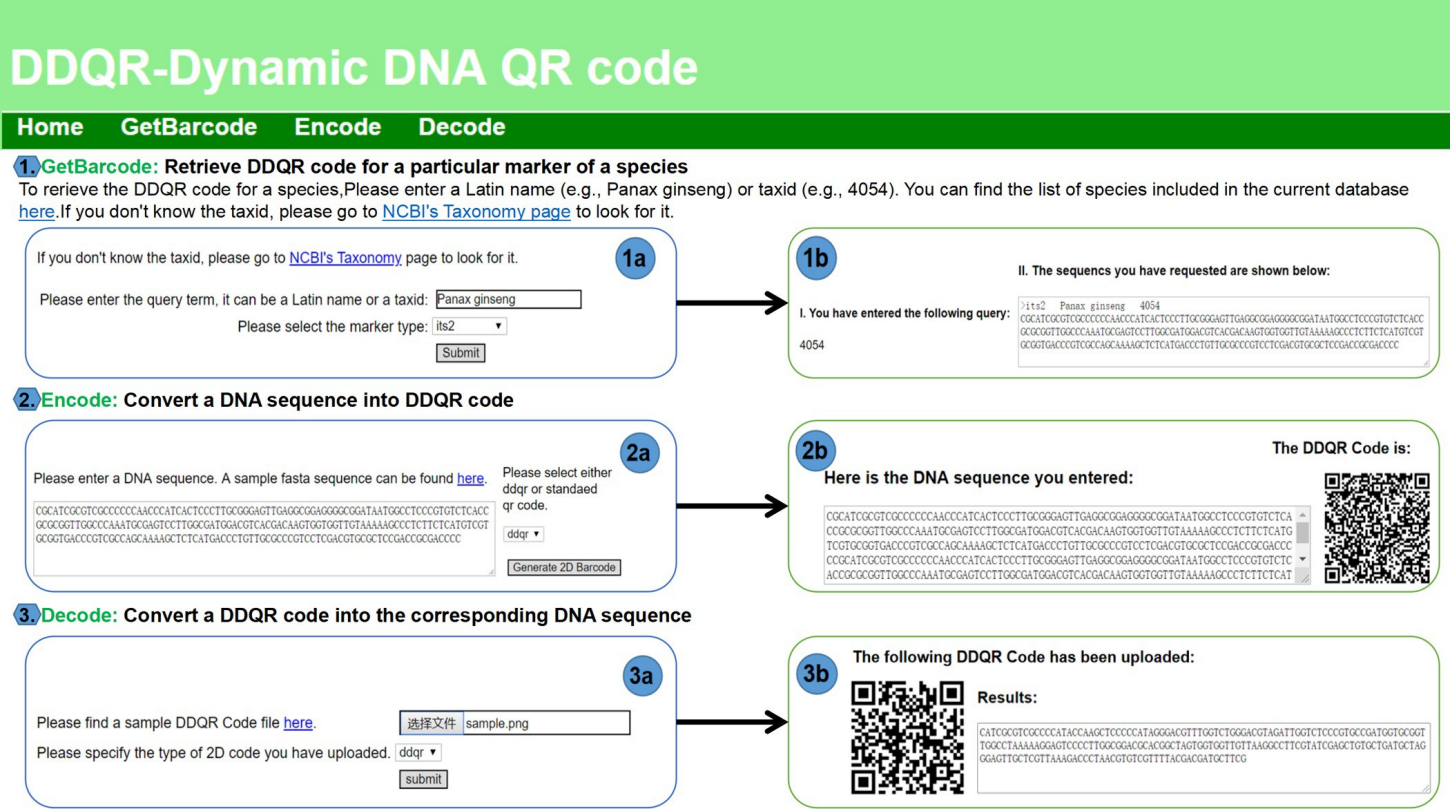
Screenshots of DDQR web server. The module numbers are shaded in blue hexagons. The module names are shown in Green. Various components on the front page, final result page, and intermediate result pages are shaded in blue circles. (1) Module "GetBarcode"; (1a) Front page for "Retrieve DDQR code for a particular marker of a species"; (1b) Results page of the module; (2) Module "Encode"; (2a) Front page for "Convert a DNA sequence into DDQR code"; (2b) Result page showing the generated 2D barcode. (3) Module "Decode"; (3a) Front page for the "Decode a DDQR code into a sequence" module; (3b) Result page showing the original DNA sequence decoded from an input DDQR code.

## 5. Discussion

In the present study, we designed and implemented a dynamic compression algorithm to improve the representation of the DNA barcode sequence as a QR code. The algorithm was compared with the state-of-art DNA compression algorithm represented by GeCo3. Our results showed that the DDQR code is more efficient in encoding short DNA sequences such as the DNA barcode sequences. However, the DDQR algorithm does not perform well compared with GeCo3 for long DNA sequences. In addition, we developed a web server that supports encoding DNA barcode sequences into DDQR code and decoding a DDQR code encoded with the DDQR algorithm.

**Fig 7:**
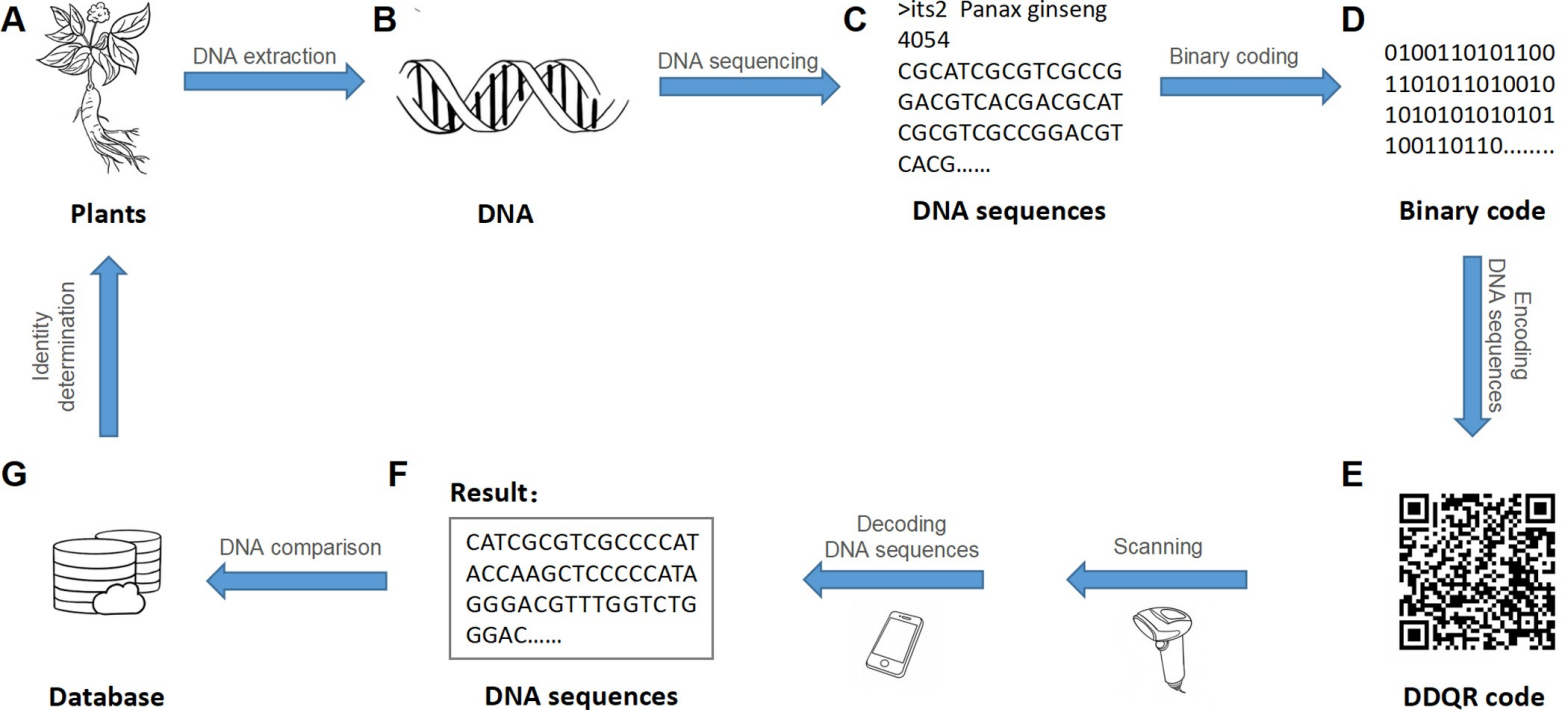
Exemplary application of DDQR in the storage and retrieval of DNA barcode sequence for plant material. We extract DNA from a plant sample (A), and the DNA sample (B) is subjected to Sanger sequencing, completing the sequence acquisition step. The resulting DNA sequence (C) is first converted to binary code (D). The binary code is then converted into DDQR code (E), completing the encoding step. Next, a scanner is used to scan the DDQR code, and then the DDQR code is decoded to the DNA barcode sequence (F). The sequence is compared with all sequences in the public database (G), allowing the identification of the plant material.

DDQR has a wide range of applications in the food and traditional medicine industries. The first application is detecting the inconsistency between the sample’s actual identity and that described in the label. The DDQR code can be used to store the DNA barcode sequence for a particular plant sample (Fig 7). Later, the DDQR code might be scanned and decoded to the DNA sequences. The DNA sequences can be used to search a public sequence database, such as GenBank, to determine the identity of the biological samples.

**Fig 8:**
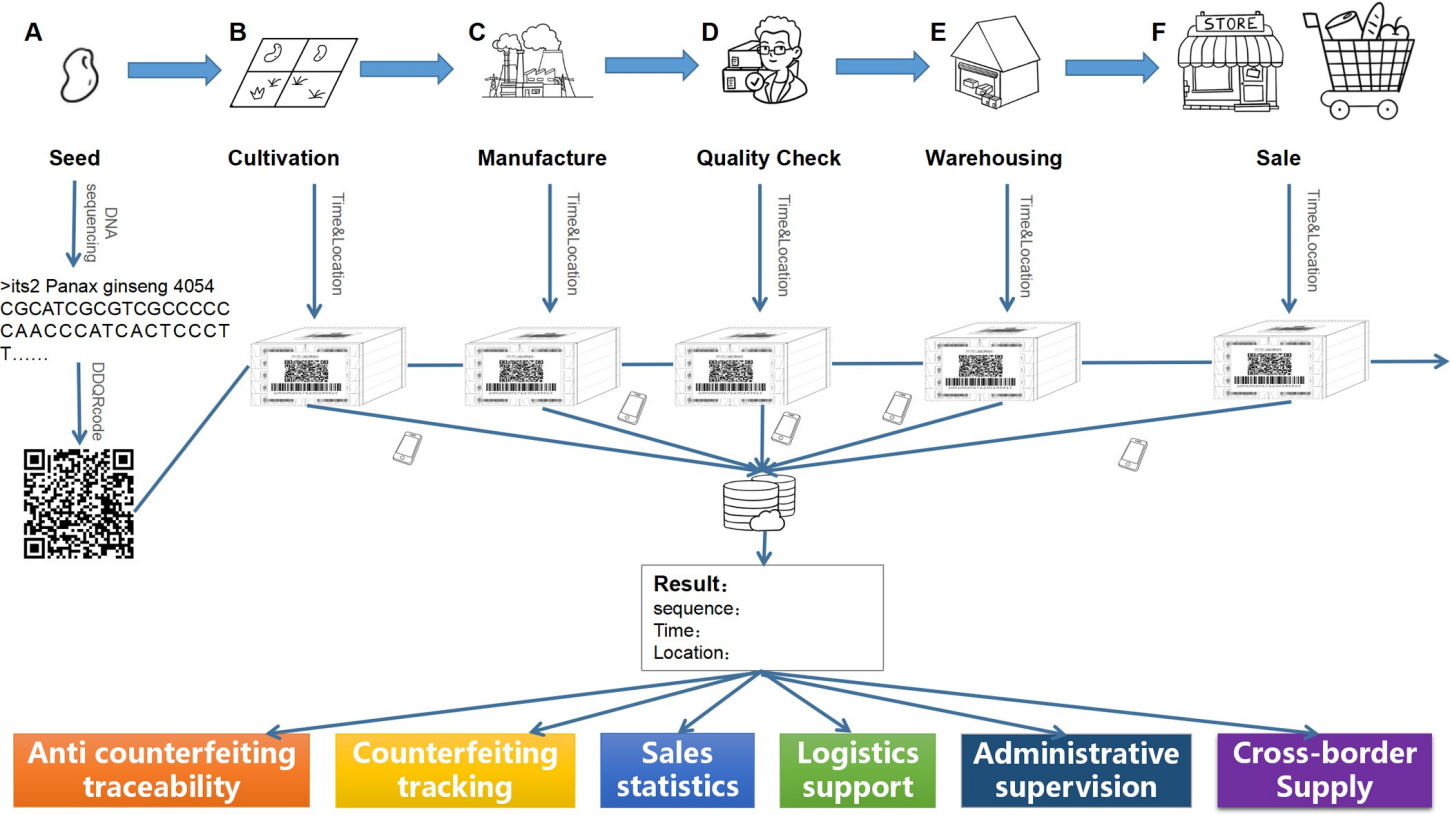
Exemplary usage of DDQR in the supply chain for various applications. Starting from a seed (A), its DNA sequence can be obtained and encoded into a DDQR code. The DDQR code would then be printed as a label that will be associated with the plant materials through the different points of the supply chains, including but not limited to cultivation (B), manufacture (C), quality check (D), warehousing (E), and sales (F). At each point, the DDQR code can be scanned. At the same time, the time and location at which the DDQR code is scanned will be recorded into a central database. The sequence, time, and location information can be retrieved to support various applications as outlined.

The second application, added to the first application, allows tracing biological materials through its supply chain. Fig 8 describes the process from the source biological materials, for example, plant seeds, to the final products reaching the consumer. The DDQR code could be obtained from the seeds at the beginning of production. And then, it can be associated with the products throughout the production and supply chain. At each phase of the supply chain, such as quality control and warehousing, once the DDQR codes are scanned, the DNA sequence, time, and location information can be stored in a central database. At any point, the DDQR code can be used as a query to search the central database to retrieve the information along the process. This system will support Anti-counterfeiting traceability, sales statistics, logistics support, administrative supervision, and cross-border supply chain management.

Several directions can be pursued in the future. Firstly, additional efficient representation might be needed. Although the DDQR algorithm has compressed the DNA barcode up to 300%, The resulting DDQR code might still be too big for real-world applications.

Secondly, additional DNA barcode sequences should be tested. With the rapid development of high-throughput DNA sequencing technologies and sophisticated informatics methods, DNA sequences at the genome scale have become affordable. For example, organelle genomes from the chloroplast and mitochondrial have been used as super DNA barcodes [13]. As a result, how to code this super DNA barcode for real-world applications will be an interesting question.

Thirdly, consideration of the reference-based method should be pursued. The mitogenome and the cpgenomes are known to be relatively conserved, at least at the genus level. It is possible to select the representative sequence from a particular taxonomic group, such as the genus at the lower taxonomic level. It will be used as references, and sequences from other species can be considered resequencing results.

Lastly, with the development of Blockchain technology, several applications have been developed to track biological products to ensure food and drug safety. The DDQR code can be used as the tag or label in the Blockchain-based tracking system. We believe the DDQR code algorithm and tools will play an essential role in food and drug safety monitoring and assurance.

## Supporting information

S1 File FASTA sequence for marker rbcL. (FASTA)

S2 File FASTA sequence for marker matK. (FASTA)

S3 File FASTA sequence for marker psbA-trnH. (FASTA)

S4 File FASTA sequence for marker ITS2. (FASTA)

S5 File FASTA sequence for marker COI. (FASTA)

S6 File Source code for DDQR and GeCo3 compression rate calculation. (PY)

S7 File Source code for DDQR compression rate calculation using simulated data. (PY)

S8 File Source code for GeCo3 compression rate calculation using simulated data. (PY)

S9 File Source code for DDQR compression rate calculation using real data. (PY)

S10 File Source code for GeCo3 compression rate calculation using real data. (PY)
